# Improved Methods for Mid-Term Blood Glucose Level Prediction Using Dietary and Insulin Logs

**DOI:** 10.3390/medicina57070676

**Published:** 2021-06-30

**Authors:** Rebaz A. H. Karim, István Vassányi, István Kósa

**Affiliations:** 1 Medical Informatics Research & Development Center, University of Pannonia, 8200 Veszprém, Hungary; rebaz.ahkarim@virt.uni-pannon.hu; 2 Cardiac Rehabilitation Institute of the Military Hospital, 8230 Balatonfüred, Hungary; kosa.istvan@med.u-szeged.hu; 3 Department of Preventive Medicine, University of Szeged, 6700 Szeged, Hungary

**Keywords:** mid-term blood glucose level prediction, basal insulin, lifestyle support for diabetes, outpatient care, artificial neural networks

## Abstract

*Background and Objectives*: The daily lifestyle management of diabetes requires accurate predictions of the blood glucose level between meals. The objective of this study was to improve the accuracy achieved by previous work, especially on the mid-term, i.e., 120 to 180 min prediction horizons, for insulin-dependent patients. *Materials and Methods*: An absorption model-based method is proposed to train an artificial neural network with the bolus and basal insulin dosing and timing, the baseline blood glucose level, the maximal glucose infusion rate, and the total carbohydrate content as parameters. The approach was implemented in various algorithmic setups, and it was validated on data from a small-scale clinical trial with continuous glucose monitoring. *Results*: Root mean square error results for the mid-term horizons are 1.72 mmol/L (120 min) and 1.95 mmol/L (180 min). The accuracy of the proposed model measured on the clinical data is better than the accuracy reported by any other currently available and comparable models. *Conclusions*: A relatively short (ca. two weeks) training sample of a continuous glucose monitor and dietary/insulin log is sufficient to provide accurate predictions. For the outpatient application in practice, a hybrid model is proposed that combines the present mid-term method with the authors’ previous work for short-term predictions.

## 1. Introduction

Diabetes is a chronic disease that presents a significant social and financial burden for a large part, ca. 9.3%, of the adult population aged 20–79 years worldwide [1]. The cause of the disease is either the lack of insulin production (Type 1, T1) or decreased insulin production combined with an acquired insulin resistance of the organism (Type 2, T2), with the latter type accounting for ca. 90% of all patients [2]. All Type 1 and more than 10% of Type 2 diabetics must rely on external insulin, most often as a subcutaneous injection applied before main meals. The dosing of these bolus insulin injections must match the diet of the patient meal-by-meal. Otherwise, too low or overly high blood glucose levels (BGL) may arise, the former (hypoglycemia) being an emergency and the latter (hyperglycemia) causing complications if it is sustained for a longer period of time. The practical consequence of these is that the majority of the patients must measure their BGL several times a day, usually before meals, using a fingertip device, and determine the insulin dosing for the meal based on the medical advice received from their doctor, the composition and amount of the (planned) meal, and their personal experience. Besides the fast-acting bolus insulin, diabetic patients also use long-acting or basal insulin injections once a day to maintain a basic, continuous insulin level for the whole day [3].

Supporting people with diabetes in insulin dosing and diet planning via information technology has gained much attention and is an active research area. The focus of our study within this wide area is the prediction of the BGL evolution between meals, using a lifestyle log (preferably integrated into a mobile app) that contains the meal composition, the insulin dosing, and the fingertip BGL measured before the meal. If a hypo- or hyperglycemia period is predicted, the patients could change the composition or quantity of the planned meal or the dosing of the insulin; thus, instead of trying to apply some general medical guidelines, they could gradually learn to manage diabetes in everyday life by example. Giving a numerical recommendation on the diet or insulin dosing is not an objective of the research.

In the following, we briefly overview the recent results and evaluation criteria of BGL prediction models. Such models are usually trained using a time series of BGL values recorded from real outpatients or from a virtual patient, i.e., an in silico model. Models trained on real data have more practical significance because even the most sophisticated virtual patient model is incapable of handling such events as changes in mental state or the environment, physical activity, stress, and so on, which do happen in a real patient’s everyday life and are known to influence the BGL evolution [4,5]. All these factors decrease the predictability of the BGL, so the accuracy of models trained and evaluated with simulated datasets is generally better than those with real patients’ data. In order to concentrate on outpatient care practice, however, we will focus on ‘real’ models in the rest of this overview.

For real patients, the BGL data for training are typically recorded over a longer time period by a continuous glucose monitor (CGM) device mounted on the patient’s body, which records the BGL computed from the serum glucose concentration every 2 to 5 min [6]. The other inputs for the training are the meals’ dietary contents, most often merely the total carbohydrate (CH) amount, and the doses of bolus insulin applied before the meals. The objective is to build a model that predicts the BGL evolution after a meal until as close to the next meal as possible (when the patient is going to apply the next insulin injection) over a prediction horizon (PH) of 30, 60, 120, or 180 min. In order to allow for the natural variations in the BGL regulation system characteristics, prediction models are usually trained and evaluated patient-by-patient [7,8,9,10,11]. An alternative approach is applying the transfer learning principle, which means that a prediction model developed for a patient is used as the startup model in the training for another patient [12,13,14,15].

The performance of the prediction models is usually evaluated by calculating the mean absolute error (MAE) or the root mean square error (RMSE), with the ‘error’ being defined as the difference between the predicted and the measured BGL at the sample points. We should note that even commonly used fingertip meters and CGM devices have an error range of ca. 1 to 3 mmol/L compared with the ‘gold standard’ intravenous measurement—the goal here is to predict the values measured by the CGM.

As a recent review on various BGL prediction methods shows, most research concentrates only on the 60 min (or even shorter) PHs [16]. However, mid-term (90, 120, and 180 min) predictions are also vital for the daily lifestyle-support of diabetic outpatients because this is the time that passes typically between two main meals. The focus of this paper is the mid-term prediction, so below, we give an overview of the nine most relevant studies in this narrower field and compare their results to the authors’ previous work, which was a direct prelude to this study.

The published studies used a wide variety of technologies for the prediction, ranging from physiological BGL regulation models through support vector regression models to neuro-fuzzy inferencing, but most of all, neural networks (NNs) of several kinds. The fundamental problem of using BGL regulation models is that, to produce reliable results, the model must be sufficiently complex, but, the more complex the model, the more parameters it has, and thus the harder it is to personalize [14,17,18]. The other methods use no BGL regulation model, only a sufficiently long BGL/lifestyle log, and artificial intelligence to learn the complex relationships between BGL and the elements of lifestyle (diet, insulin, and even other factors such as stress or physical activity). If the patient wears a CGM all the time and follows a regular daily meal schedule, the model may be based purely on the CGM and, optionally, insulin data, without any dietary information. The latest result of this kind was presented by Kushner et al., who developed a feed-forward NN (FNN)-based model for 24 patients with good results on the mid-term PHs (RMSE 120 min: 2.11, 180 min: 2.2 mmol/L) [14]. A similar prediction accuracy (120 min: 2.08 mmol/L) was reached by Zarkogianni et al., earlier in 2015 with an adaptive neuro-fuzzy system, though the 10-person study also used information on physical activity [9]. A similar study by Montaser et al. reached much better results in 2020 (60 min: 1.04), though on patients using closed-loop CGM-insulin pump devices, where the BGL variations are generally smaller [19]. Aliberti et al. (2019) and Frandes et al. (2016) followed the purely CGM-based approach on auto-regressive NN structures [8,12]. The 90 min RMSE results are 1.59 and 1.23 mmol/L on 451 and 17 patients, respectively, but no information is available for longer horizons.

The earlier work of El Georga et al. is particularly relevant for this review because, like Kushner et al., they also consider in the prediction the diet and, additionally, the physical exercise, with the latter measured by non-invasive devices [10]. The model was trained and evaluated for two patients with 5/11 days of data with a 120 min RMSE of 1.88 mmol/L. Significantly worse results of this kind are reported by Mathiyazhagan (2014) [20].

Finally, in a previous study, the authors proposed to use a simple absorption model to calculate parameters related to CH absorption dynamics (like the time to peak) to train an FNN instead of the ‘raw’ CH value used for training in previously published studies [21]. The idea was that, because the slope of the absorption curve is known to depend on other parameters of the meal like fat or fibre content besides the CH content, the extra information conveyed by these parameters could lead to more accurate predictions, even with a smaller set of training data that does not contain all food composition varieties. The results confirmed this hypothesis, and good accuracy was achieved, especially on the 60 min PH. As that work was the starting point of the current study, it will be referred to as the pre-study in this paper. Table 1 summarizes the key parameters and results of the models described above. For a more detailed review of several more studies, please see [21].

**Table 1:** Recent results on high blood glucose level (BGL) prediction models on real patients, for 60 min or longer prediction horizons (PHs). Authors’ previous study in italics at the bottom. N/A: no data available.

Author and Year	Method and Model Inputs	Patients and Training Data Sets	Results RMSE [mmol/L] for each PH
Kushner 2020 [14]	FNN, shallow neural networks Inputs: CGM, insulin	24 patients, duration: N/A	90 min: 1.83
			120 min: 2.11
			180 min: 2.22
			240 min: 2.39
Montaser 2020 [19]	Seasonal autoregressive integrative moving average Inputs: CGM, insulin, and energy expenditure	18 patients with closed-loop CGM/insulin pump, duration: 60 h	30 min: 0.57
			60 min: 1.04
			90 min: 1.24
Liu 2019 [7]	Physiological model Inputs: CGM, CH	10 patients, duration: 14 days	60 min: 1.68
			90 min: 2.12
			120 min: 2.25
Aliberti 2019 [12]	Non-linear autoregressive NN Inputs: CGM only	451 patients, duration: more than 2 days	60 min: 0.73
			90 min: 1.59
Frandes 2016 [8]	Auto-regressive NN Inputs: CGM only	17 patients, duration: 4–7 days	30 min: 0.13
			60 min: 0.24
			90 min: 1.23
Zarkogianni 2014–2015 [9,22]	Adaptive neuro-fuzzy inference Inputs: CGM, physical activity	10 patients, duration: 6 days	60 min: 1.26
			120 min: 2.08
Mathiyazhagan 2014 [20]	Adaptive network-based fuzzy inference system Inputs: CGM, insulin, and CH	2 patients, duration: 52 days	30 min: 1.72
			60 min: 3.16
			120 min: 5.71
El Georga 2010 [10]	Support vector regression Inputs: CGM, insulin, and CH	2 patients, duration: 5, 11 days	60 min: 1.28
			120 min: 1.88
Finan 2009 [23]	Autoregressive moving average Inputs: CGM only	6 patients, duration: 2–8 days	30 min: 1.50
			60 min: 2.50
			90 min: 3.39
Pre-study 2020 [21]	FNN Inputs: CGM, insulin, and glucose absorption curve from model	5 patients, duration:1 1–23 days	60 min: 1.12
			90 min: 1.62
			120 min: 1.76
			180 min: 2.18

**Figure 1:**
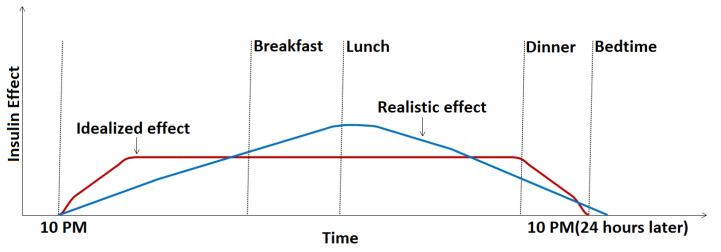
Ideal and realistic basal insulin effect in the function of time.

The motivation for the current work is that, although the model proposed in the pre-study delivered very good results for the 60 min PH, the mid-term accuracy was much worse. The hypothesis of the current study is that the mid-term inaccuracy in the pre-study was due to two factors:

- The model does not account for the slowly weakening effect of the basal insulin. Ideally, basal insulin should very quickly produce a constant base insulin effect that is maintained for 24 h and drops sharply to zero by the time of the next basal injection, but the build-up and cessation of the effect are, in fact, gradual [3], similar to that shown in Figure 1. As basal insulin adds to the bolus insulin, we can thus expect that the same bolus amount will have less of an effect for a meal at noon than in the evening before applying the next basal dose. Thus, if basal insulin information could be included in the prediction model inputs, we expect that the model could better adapt to this slowly changing effect.
- The pre-study used absorption parameters that characterized only the first one hour of the absorption process. Mid-term (120 and 180 min) predictions are expected to benefit from adding more information about the process’s whole duration.

The objective of the current work can thus be defined as finding a prediction method that surpasses the pre-study and other published methods on the longer horizons by including the above effects in the prediction model.

## 2. Methods

### 2.1. The Artificial Neural Network

**Figure 2:**
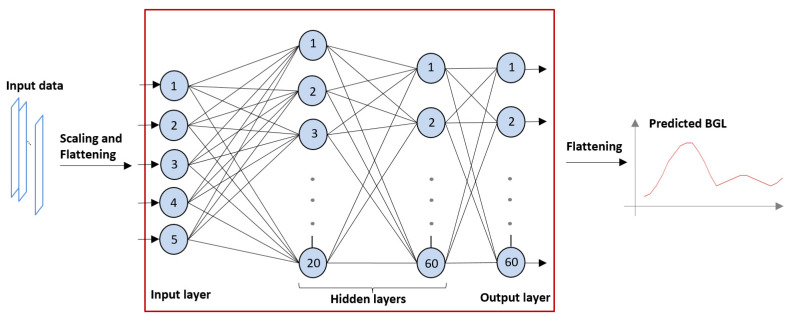
Structure of the feed-forward neural network (FNN) used for prediction. Numbered circles represent neurons organized in layers.

The technology used to develop the prediction model was a feed-forward artificial neural network. An FNN contains a network of processor nodes (each resembling the functionality of a human neuron), connected in layers, each layer feeding the next layer and the last layer producing the outputs; in our case, the predicted BGL values. The neurons of the input layer of the FNN receive the inputs; in our case, the parameters derived from the meals and the insulin dosing. Every neuron has several inputs from the previous layer, and it computes its output from its weighed inputs using an activation function. The prediction model in the weights of the inputs (synaptic weights) of each neuron. Figure 2 shows the structure of the FNN used in our study. The value range of all inputs should be similar, so the inputs should be scaled, and the input/output vectors must be rearranged from/into a time series of data (flattening).

In order to learn the behavior of the complex system being modeled, the network is trained in several iteration cycles, using the samples from the training set. The training is performed according to the following steps:

1. The FNN is initialized with a set of default weights;
2. The expected output, in our case, the recorded BGL values, is loaded in the nodes in the output layer, comparing them to the current output, and computing the error;
3. The error is used in the underlying layers to change the synaptic weights according to a ‘learning’ regime [24];
4. This process is iterated several times. The training is successful if the error is gradually decreasing across several iterations, i.e., the network converges;
5. The next input/output training sample is loaded into the FNN, and the training is continued with the final synaptic weight set of the previous training sample as the startup weight set.

The training ends when the processing of the last training sample is complete, thus every sample can exert its effect on the synaptic weights.

The number layers and neurons in the layers in an FNN must match the application. As the CGM device used in the trial took a measurement every 2 min, the number of outputs in our study was set according to the horizon: 30 outputs for the 60 min, 45 for the 90 min, 60 for the 120 min, and 90 for the 180 min prediction. Figure 1 shows a 120 min arrangement.

**Figure 3:**
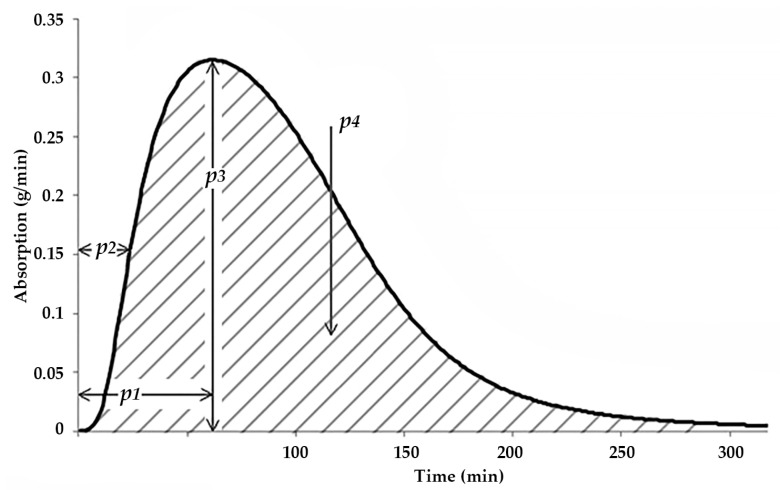
Parameters of the estimated glucose absorption rate curve.

- The FNN has some algorithmic parameters that must be tuned for a particular application and training set in order for the network to converge. In our survey, these parameters were determined empirically to achieve the best results as follows.
- The quasi-Newton method was used as the training regime [25];
- The neurons’ activation function was set to hyperbolic tangent, a smooth transition function used most often for NN training;
- For the learning regime, a method faster than traditional back-propagation, the Brent training rate method, was used with a training tolerance rate of 0.000001 [26];
- The number of hidden layers was set to 2, the first layer containing 20 neurons and the second containing 60 (as shown in Figure 1);
- The maximum number of iteration cycles was set to 118. Using more hidden layers or more iteration cycles was found to result in over-training (the model was too specific for the training sample), producing worse predictions;
- In order to transform the value ranges of the inputs into a common range, scaling was performed for two of the input parameters via division by 100 or 1000 (see the proposed input list below Figure 3);
- The error threshold was set to 10 × 10^−16^, i.e., an error below this threshold terminated the training process.

The FNN implementation used for the study was the publicly available OpenNN C++ library [27]. The training was executed on a Dell Studio XPS 8100 computer (Intel Core i7 2.93 GHz CPU, 8 MB cache, Quad-Core; 16 GB RAM) with a 2 GB NVIDIA Quadro K2000 graphics, and the training process took around 3–16 min for each patient and each prediction horizon, depending on the horizon.

### 2.2. The Inputs of the FNN

The core idea of this study is to add new FNN input parameters to the original set used in the pre-study in order to include information on the longer-term BGL related processes. The original five parameters were applied: bolus insulin dose (BI), the startup (baseline) BGL (SBGL), and three more parameters related to the estimated dynamics of the absorption of the meal. These latter parameters were computed for each meal using a simple, but powerful two-compartment absorption model from Arleth et al. The Arleth model can estimate the absorption curve during the digestion of a meal based on the consumed quantities of proteins, lipids, dietary fibers, monosaccharides, and starch. As the absorption curve shows the estimated rate of glucose infusion into the blood as the function of time, it is a more precise source of information than just the total CH content of the meal. For more details on the absorption model, please see [28,29]. The effect of the glycemic parameters on glucose absorption is exemplified, and the pre-study model is described in more detail in [21].

Figure 3 shows the absorption-related parameters used in the pre-study, i.e., the time to the peak of the absorption curve (TPeak, p1 in Figure 3), the time to 50% of the peak (T50, p2 in Figure 3), and the maximal rate of CH absorption (MaxCh, p3 in Figure 3).

In order to improve mid-term accuracy, we propose the following two extra parameters:

- The area under the whole absorption curve (AuC, p4 in Figure 3). This parameter is expected to describe the longer-term effect of the current meal;
- The time elapsed since the application of the last basal insulin injection (DfB). This parameter is expected to exert a smaller, but positive effect in all meals.

While training the model, our experimental results with various algorithmic setups and neuron numbers, among others, showed that we had to limit the number of FNN inputs to 5, as 6 or more inputs would make the convergence of the FNN very slow or stop it altogether. This effect is the result of the limited size of the training data set available for each patient (see next subsection)—too many features and a small set of training samples prohibit the successful training of a neural network [30]. Therefore, we kept only a single important dynamic parameter, MaxCH, and included the two new ones, as described above. MaxCH, the maximal absorption rate, was selected because, by common sense, this carries the most information on the shape of the absorption curve in the 60–180 min interval (when combined with the area under the curve parameter), assuming that the time to peak is within or close to 60 min. In summary, the proposed new input set is as follows.

1. BI: The applied bolus insulin dose, in [pmol/1000];
2. SBGL: The startup BGL, in [mmol/L];
3. MaxCH (p3 in Figure 3): The maximal rate of CH absorption, in [g/minute];
4. AuC (p4 in Figure 3): The area under the absorption curve, in [g];
5. DfB: The time elapsed since the last basal insulin, in [minute/100].

To distinguish this strategy from the original absorption-based strategy code-named ABS in the pre-study, we will refer to it as AUC-DFB, i.e., area under the curve and delay from basal.

As the new parameter set does not contain information on the first part of the absorption process before the peak, it can be expected that the short term (60 min or less) accuracy of the model will decrease, especially because the average time to the peak of all estimated absorption curves is 59 min, i.e., ca. 1 h.

Therefore, a hybrid (combined) model is proposed in which we use the ABS model for horizons shorter than or equal to 60 min and the AUC-DFB for 90 min or longer predictions.

### 2.3. The Clinical Trial Protocol

The data were collected in a small-scale clinical trial running in 2019 at the Cardiac Rehabilitation Institute of the Military Hospital, Balatonfüred, Hungary. For details on the informed consent and the ethical approval of the trial, please see the Declarations section. As the patients took part in rehabilitation after a cardiac disease, they led a lifestyle quite close to their everyday life for several days. The rehabilitation involved moderate, but regular (daily) physical exercise, and the patients lived in a quiet and relaxing environment. The continuous medical supervision and the controlled meals made it very easy to collect and manage the source data. The patients kept a paper-based diary that contained wake-up/bedtime, insulin administration, physical activity, and meals as events. Basal and bolus insulin types and doses were distinguished. The meals were designed by the dietitians of the hospital; however, the patients had a choice from several options. These choices and the estimated quantity (like ‘one slice of bread’) were recorded in the diary by the patient. The patients wore a CGM device for the full duration of the trial that was calibrated daily using fingertip BGL measurements. The baseline fingertip BGL readings before the meals were also recorded in the diary. At the end of the trial, the diaries were collected and checked for completeness.

### 2.4. Data Used for Training and Validation

The clinical data set was the same as that used in the pre-study. Three patients out of the original eight were excluded owing to the incompleteness of their dietary and insulin log. Table 2 summarizes the characteristics of the source data. We excluded those meals for which no insulin was applied before the meals, those with a total CH content of less than 5 g (irrelevant meals), and those that preceded another meal within 60 min.

We could use the same clinical data for this study as used in the pre-study, with the exception of the daily basal insulin injections. These data items were recovered from the paper diaries for this study. After these steps, the original set of 365 meals for the five patients was reduced to 167 meals, distributed among the patients as shown in Table 2. Four patients suffered from T2 and only one (P05) from T1 diabetes.

### 2.5. Training and Validation Methods

The CGM samples for each patient were divided into two parts; two-thirds were used to train the FNN and one-third for validation, e.g., for the patient P02, the total 43 meals were divided as 28 meals for training and 15 for validation. The division between the training and validation set was made according to the date and time of the meals.

To measure the performance of the prediction, we computed the RMSE and MAE for each PH and each patient, using the model generated specifically for the patient. We also computed the RMSE and MAE for all validation CGM samples from all five patients.

The robustness of the generated models with respect to training/validation data separation was checked via threefold cross-validation, for the mid-term horizons. For each patient, two more models were trained using another third of the samples for validation, and the performances of these models were compared to that of the original.

The efficiency of FNN models is known to depend on the size of the training set, and there were considerable differences in the number of meals available (ranging from 43 for P02 to 26 for P03), which can add a bias to the patient-wise analysis of the results. To check the effect of the training set size and separate this effect from other effects, we repeated the training process for the P01, P02, P04, and P05 patients such that the number of meals was limited by random selection to 26, of which 17 were used for training and 9 for validation.

Finally, we also implemented and tested two more versions of the proposed AUC-DFB strategy to assess the effect of the proposed two new parameters, the AUC and DFB versions as follows:

- ‘AUC’ version: the TPeak parameter is used instead of the DfB parameter;
- ‘DFB’ version: the TPeak parameter is used instead of the AuC parameter.

Table 3 summarizes the input parameter sets of the above versions.

The assessment was performed by systematically comparing the RMSE and MAE of the AUC, DFB, AUC-DFB, and ABS versions (the latter from the pre-study). RMSE differences between the AUC-DFB and the ABS versions were checked by the paired sample *t*-test.

### 2.6. Medical Devices and Data Processing Tools

The Medtrum’s S7 EasySense CGM System was used in the clinical trial, which registered BGL values every 2 min [31]. Mérykék 800 fingertip BGL sensors were used to calibrate the CGM device every day and to record the baseline BGL before meals. Any deviation from the controlled meals was recorded by the patients on paper forms.

The dietary and insulin logs were recorded in a MongoDB database [32]. The ingredient quantity and the GI values for the absorption model were computed from the dietary logs using a dietary expert database developed earlier by the authors’ research group [33]. The database contains the dietary composition of the foods and dishes commonly consumed in Hungary.

The absorption model was implemented in the C++ programming language according to the original paper of Arleth [29], in the form of a research-oriented desktop application [34]. Microsoft Excel 2013 was used for statistical analysis and visualization.

## 3. Results

### 3.1. Accuracy Results of the Various Model Versions

Table 4 shows the accuracy achieved with the AUC-DFB strategy for the five patients separately and for all datasets.

The effect of the two new parameters was evaluated in the versions AUC and DFB. Table 5 and Table 6 show the results of these tests.

The AUC and DFB models benefited from an additional dynamics parameter, the time to peak of glucose absorption. The accuracy is less on mid-term PHs in these versions compared with the AUC-DFB, although AUC is very close.

### 3.2. Performance of AUC-DFB Compared with the Pre-Study and the AUC/DFB Versions

The RMSE differences between the new AUC-DFB and the original ABS version proposed in the pre-study are shown in Table 7. Note the significantly worse results of the new version compared with the ABS on the 60 min, and its improvement over the ABS on the 180 min PH. In this comparison, we used the predicted values from all patients, from the whole time range of the PH, e.g., values for 90 time instants for the 180 min horizon (due to the 2 min sampling interval of the CGM).

If we construct a hybrid model from the ABS and AUC-DFB models such that, in the first 60 min of the prediction window, we use the ABS prediction and, after that, the AUC-DFB prediction, then it makes sense to compare the mid-term performance of the two models using only the 60 to 180 min time frame. The comparison in Table 8 was made separately for the 60 to 120 and 120 to 180 min performance of the two models. The improvements are more convincing than those in Table 7 because the poor performance of AUC-DFB in the first 60 min has no effect on the result. Nevertheless, it can be argued that this is the real improvement that users experience when they switch to using the hybrid model instead of the ABS, i.e., using the proposed AUC-DFB for the second and third hour. Thus, in terms of RMSE error, we can summarize the accuracy of the hybrid model as 0–60 min: 1.12 mmol/L, 60–120 min: 1.66 mmol/L, and 120–180 min: 1.83 mmol/L.

Table 9 compares the performance of the AUC-DFB model to the variations in AUC and DFB to assess the effect of the proposed two new parameters separately.

**Figure 4:**
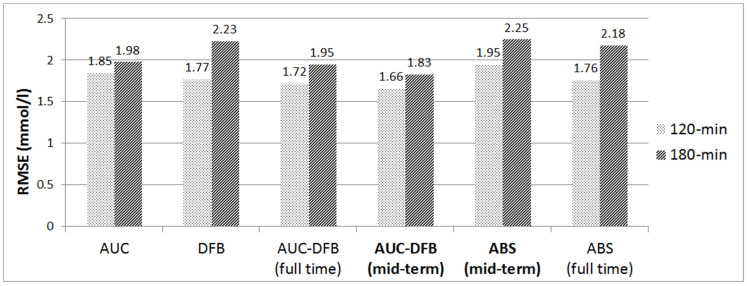
RMSE results on the 120 and 180 min PHs, comparison of the various prediction models. All values are in mmol/L.

Figure 4 graphically summarizes the mid-term performance of the prediction model versions.

### 3.3. Accuracy Results of Cross-Validation and with Limited Data Set Size

Threefold cross-validation was used to check the robustness of the data selection scheme for training and validation records for the proposed AUC-DFB version (Table 10). Only the 120 min and 180 min PHs were evaluated because the AUC-DFB is proposed only for mid-term PHs, i.e., above 60 min. As the table shows, the accuracy differences are within 10%.

Finally, we measured the three new versions’ performance with a limited data set size (that of P03) used for the training and validation for all patients. As could be expected, the measured accuracy values are worse than the original ones shown in Table 4 for all patients (except P03, naturally). The AUC-DFB RMSE results on the 180 min horizon are as follows: P01 3.203 vs. 3.080, P02 2.014 vs. 1.542, P04 1.627 vs. 1.449, and P05 1.922 vs. 1.789. For the detailed results on all horizons and versions, please contact tha authors.

## 4. Discussion

### 4.1. Comparison of Model Training Versions

As Table 4, Table 5 and Table 6 show, the proposed AUC-DFB version outperforms the AUC and the DFB ones, especially on the longest (180 min) PH, confirming the hypothesis that the new parameters, i.e., the basal insulin timing and the total CH content, both carry essential information about the long term BGL evolution, though the AUC-DFB vs. AUC difference is small (1.80%, see Table 9). This fact and the direct DFB-AUC comparison in the middle section of Table 9 show that the effect of the basal insulin timing on BGL is less than that of the total CH content—possibly owing to the relatively low quantity of basal insulin compared with the bolus insulin doses.

The comparison of the performance of the new AUC-DFB version to that of ABS (proposed in the pre-study) in Table 7 and Table 8 shows that, in line with our expectations, we could improve the mid-term performance only at the expense of the short-term (60 min) performance. Therefore, in a practical application, both models should be used for an accurate 0 to 180 min prediction (the ‘hybrid’ model).

### 4.2. The Effect of Data Set Size and Selection 

The accuracy data in Table 4, Table 5 and Table 6 show considerable variations among the patients, e.g., on the 180 min PH 3.08 mmol/L for P01 versus 1.542 mmol/L for P02. Such big differences may be caused by different training sample sizes (29 vs. 43 meals, see Table 2), but the results of the training sessions with the same sample size show that there are still considerable, though generally smaller differences—thus the effect cannot be explained by the sample size alone. The explanation may be the different quality of the patients’ dietary log or some other effects that our study could not measure, like the strength of the effect of emotional state on the BGL, which can typically vary with the patients, making one patient more ‘predictable’ than the other.

As the cross-validation tests (Table 10) found only minor differences among the models, we can conclude that the training/validation data division does not play an important role concerning prediction performance; in other words, the data were quite homogeneous even with such small sample sizes.

### 4.3. Clinical Significance of the Improvement

The achieved 0.3–0.4 mmol/L improvement in the accuracy over the previous mid-term results is less than the error range of the CGM devices. Naturally, we used the fingertip-calibrated CGM as reference data for practical reasons (applicability in a realistic clinical trial), similarly to several other studies, with the primary objective being the methodological improvement of BGL prediction and not that of measurement technology. In other words, the same proposed prediction method could be used with any other reference BGL data. If we can improve the accuracy of the prediction method, then the overall accuracy of the prediction compared with the gold standard will also improve. We can also assume that the accuracy of CGM (and other similar noninvasive devices) will improve with the advancement of the measurement technologies, improving the overall accuracy of the prediction.

### 4.4. Comparison of the Results to Results Published by Others

When the results of several alternative methods are compared numerically for assessment, an ideal comparison scheme would require that the various methods be run on the same input data. Unfortunately, data sets are seldom published in the literature, for various reasons. Another more fundamental problem that renders predicted value-by-value comparison impractical is that the methods use heterogeneous input parameter sets that are often specific to the clinical trial. Our source dietary log data, for example, could not be reproduced from a trial that logged only raw CH values. Therefore, the discussion below must be limited to the comparison of the measured accuracies.

The number of patients in the reviewed studies varies between 2 and 451 (Table 1), but most of them, including ours, can be classified as ‘small-scale’ trials. This may be due to the problems associated with recruiting volunteers for CGM-based protocols. However, because, in most cases (including our approach), a model trained for a certain patient has no effect on the model of another patient, it is not the number of patients, but the length of the training/validation data sets for each patient, that limits the performance attainable by the model. A higher patient number would allow the analysis of natural variability in the ‘predictability’ of patients—with five patients in our cleaned data set, this was not a goal of our survey. In our proposed application scenario (see below), the patient runs their own personalized model on their own device, and the accuracy of the predictions can always be measured empirically and individually, so the applicability of the proposed method does not depend on large-scale trial results.

As it is the original ABS model that we propose for the 60 min PH and that model was evaluated and compared to related work in the pre-study, here, we focus on mid-term PHs only.

Four relevant studies listed in Table 1 use no dietary data for the prediction, of which two rely solely on CGM (Aliberti and Frandes), and the other two (Kushner and Zarkogianni) combine CGM with insulin dosing or physical activity information. As glucose infusion into the blood is a key input of the BGL regulation system, the lack of dietary data for model training allows only two alternatives:

- either the patient has to wear a CGM all the time (in which case the model can identify a typical BGL excursion in real time based on past CGM patterns);
- or the contents and daily scheduling of the patient’s meals must be very similar (in which case the model can assume that similarly scheduled BGL patterns will appear every day).

These limitations make it hard to compare their results to those studies of those of this study directly, especially because Aliberti and Frandes do not have results for longer PHs above 90 min. The 90 min results of these two studies are better than those of AUC-DFB, but on longer horizons, AUC-DFB performs better. The 120 min RMSE reported by Kushner and Zarkogianni (2.11 and 2.08 mmol/L) compares favorably to that achieved by the AUC-DFB model (1.72). A 180 min result is only available for Kushner of the above four authors: 2.22 mmol versus 1.95 of AUC-DFB.

El Georga (2010) and Liu (2019) took an approach similar to ours by relying on CH content, though without absorption dynamics or basal insulin information. The 120 min result of El Georga, tested on only two patients, is only slightly worse (1.88 mmol/L) than AUC-DFB, while the most recent study of Liu, with 24 patients, falls behind more considerably (2.25 mmol/L). Unfortunately, 180 min results are quite rarely published in the literature, so we cannot compare AUC-DFB to these models on this PH.

**Figure 5:**
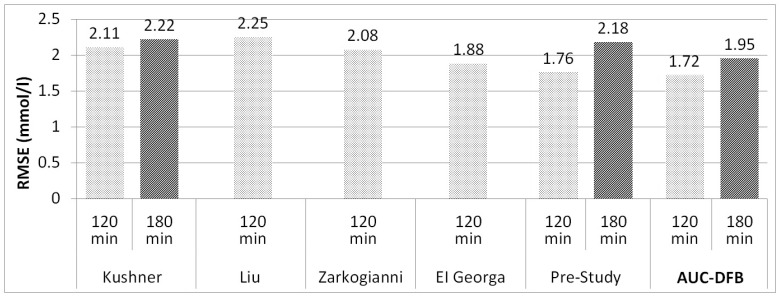
RMSE results on the 120 and 180 min PHs, comparison of related work, and the pre-study to current work (AUC-DFB). The 180 min results are not available for all studies. The 120 min result of Mathiyazhagan of 5.71 mmol/L was not included in the figure to preserve a readable vertical scaling of the chart. All values are in mmol/L.

As this study’s primary focus is improving the accuracy on mid-term PHs, Figure 5 shows a graphical summary of the 120 and 180 min results. As the time frame extends, the advantages of including long-term parameters in the model training process appear.

### 4.5. A Proposed Application Scenario

In the proposed application scenario, the patient wears a CGM for two weeks or 50 meals while logging their meals and insulin dosing with a mobile, interactive lifestyle support tool [35]. Insulin logging can even be made automatic using modern insulin pens with a wireless connection to the app [36]. Based on these data, the ABS and AUC-DFB models are trained and validated within the same lifestyle support app, and the patient can get reliable short- and mid-term BGL predictions from the app without relying on CGM anymore. Training the ABS and AUC-DFB models in parallel would pose no particular technical difficulty, though it is the mid-term prediction that has more clinical significance if our goal is to reach a safe BGL by the time of the next meal. This scenario is more realistic than one based on wearing a CGM for an indefinite period of time. For a large part of insulin-dependent patients, continuous CGM use is not feasible for financial reasons and because of the inconvenience of wearing even the most sophisticated CGM device all the time. Nevertheless, if data from later CGM sessions become available, the models can be refined separately, or a single model with more inputs could be trained.

### 4.6. Limitations of the Study

The number of patients varies between 2 and 451 (Table 1) in the reviewed studies, but most of them, including ours, can be classified as ‘small-scale’ trials. This may be due to the problems associated with recruiting volunteers for CGM-based protocols. However, because, in most cases (including our approach), a model trained for a certain patient has no effect on the model of another patient, it is not the number of patients, but the length of the training/validation data sets for each patient that could limit the real performance attainable by the model. A higher patient number would allow the analysis of natural variability in the ‘predictability’ of patients—with five patients in our cleaned data set, this was not a goal of our survey. In our proposed application scenario (see above), the patient runs their personalized model on their own device, and the accuracy of the predictions can always be measured empirically and individually, so the applicability of the proposed method does not depend on large-scale trial results.

Another limitation of the results’ applicability is that, because of the rehabilitation setting of the clinical trial, the effects of more intensive mental or emotional stress and random (irregular) physical exercise could not be modeled in our study. Contrary to our design, most of the referenced studies, especially the CGM-only protocols, involved free living outpatients, with regular sessions at the clinic in some cases [19]. However, we think that the other differences in the study designs, like the absence of dietary information, were more important factors in the comparison of the results than the effect of the rehabilitation versus free living setting. In our case, this latter effect could be measured in the future by the same protocol extended by further monitoring the same patients in the same way after leaving the hospital.

## 5. Conclusions

The paper presented a method for improving the mid-term BGL predictions for people with diabetes. The main contribution is the use of longer-term parameters, like the basal insulin timing, for the training of an FNN. The approach was validated on real data from a small-scale clinical trial. The importance of the results is due to the fact that the first 60 min after a meal, on which most of the previous research (including the authors’ previous work) is concentrated, is only one-third, or less, of the time normally elapsed between two main meals of a patient with diabetes. The proposed new AUC-DFB training scheme resulted in a significant improvement in the RMSE accuracies on the 120 and 180 min horizons, surpassing those of any other similar study published to date, to the authors’ best knowledge (60–120 min: 1.66 mmol/L, 120–180 min: 1.83 mmol/L). For the outpatient application in practice, a hybrid model is proposed that combines the AUC-DFB mid-term method with the authors’ previous work (ABS) for short-term predictions. Future work should focus on tracking the effect of other factors like stress and physical activity.

## Figures and Tables

**Table 2:** Source data summary (data available for the study after data cleaning).

	P01	P02	P03	P04	P05	Sum
Gender	Female	Female	Male	Female	Male	-
Age	52	49	33	18	23	-
Height	169	175	160	183	197	-
Weight	77	133	50	97	82	-
Diary long (days)	24	23	15	12	15	89
Number of meals	29	43	26	34	35	167
Breakfast	8	16	7	10	7	48
Lunch	9	17	8	9	7	50
Dinner	6	10	5	11	8	40
Other	6	0	6	4	13	29
Number of insulin injections	38	62	35	44	45	224
Number of CGM records	3480	5160	3120	4080	4200	20,040

**Table 3:** Input parameters of the pre-study and the three new versions.

Version	Parameters				
Pre-study (ABS)	BI	SBGL	MaxCH	TPeak	T50
AUC	BI	SBGL	MaxCH	AuC	TPeak
DFB	BI	SBGL	MaxCH	TPeak	DfB
AUC-DFB	BI	SBGL	MaxCH	AuC	DfB

**Table 4:** Root mran square error (RMSE) and mean absolute error (MAE) results for the five patients with the area under the curve and delay from basal (AUC-DFB) version on four horizons, all values in mmol/L.

Patient	Figure of Merit	60 min	90 min	120 min	180 min
P01	MAE	1.892	1.895	1.985	2.595
	RMSE	2.165	2.156	2.317	3.080
P02	MAE	0.939	1.098	1.105	1.320
	RMSE	1.107	1.338	1.398	1.542
P03	MAE	1.181	1.366	1.645	1.621
	RMSE	1.761	1.950	2.164	2.246
P04	MAE	1.050	1.048	1.058	1.132
	RMSE	1.171	1.193	1.241	1.449
P05	MAE	1.127	1.280	1.359	1.402
	RMSE	1.499	1.717	1.756	1.789
All datasets	MAE	1.201	1.304	1.470	1.562
	RMSE	1.486	1.624	1.718	1.946

**Table 5:** RMSE and MAE results with the AUC version on four horizons, all values in mmol/L.

Patient	Figure of Merit	60 min	120 min	180 min
P01	MAE	1.844	2.074	2.681
	RMSE	2.048	2.479	3.152
P02	MAE	0.825	1.233	1.325
	RMSE	1.031	1.521	1.609
P03	MAE	1.128	1.914	1.548
	RMSE	1.624	2.445	2.256
P04	MAE	0.888	0.990	1.217
	RMSE	1.036	1.228	1.419
P05	MAE	1.094	1.534	1.433
	RMSE	1.456	1.908	1.839
All datasets	MAE	1.116	1.579	1.591
	RMSE	1.388	1.850	1.981

**Table 6:** RMSE and MAE results with the DFB version on four horizons, all values in mmol/L.

Patient	Figure of Merit	60 min	120 min	180 min
P01	MAE	1.718	2.067	2.602
	RMSE	1.954	2.432	3.415
P02	MAE	0.842	1.170	1.434
	RMSE	1.038	1.413	1.805
P03	MAE	1.078	1.643	2.023
	RMSE	1.604	2.243	2.594
P04	MAE	0.747	1.026	1.383
	RMSE	0.891	1.286	1.655
P05	MAE	1.059	1.465	1.607
	RMSE	1.363	1.806	2.103
All datasets	MAE	1.055	1.505	1.750
	RMSE	1.321	1.773	2.233

**Table 7:** RMSE comparisons between the ABS model and the AUC-DFB model using all predicted values in the range of the horizon, i.e., all predictions between 0 and 180 min for the 180 min prediction. A negative difference means that the error increased. All values are in mmol/L.

	AUC-DFB	Pre-Study (ABS)	Diff. Value	%	*t*-Test
60 min	1.486	1.12	−0.366	−32.69%	*p* = 0.0332
120 min	1.718	1.76	0.037	2.14%	*p* = 0.0524
180 min	1.946	2.18	0.23	10.59%	*p* = 0.0033

**Table 8:** RMSE comparisons between the ABS model and the AUC-DFB model using only the predictions that fall into the time range shown, i.e., predictions between 60 and 120 for the 120 min prediction and predictions between 120 and 180 for the 180 min. All values are in mmol/L.

	AUC-DFB	Pre-Study (ABS)	Diff. Value	%	*t*-Test
60 to 120 min	1.655	1.947	0.292	14.99%	*p* = 0.0272
120 to 180 min	1.827	2.253	0.426	18.89%	*p* = 0.0147

**Table 9:** Differences between the performance of model variations. A negative difference means that the error increased. All values are in mmol/L.

			Prediction Horizon					
			60 min		120 min		180 min	
			MAE	RMSE	MAE	RMSE	MAE	RMSE
AUC-DFB Compared to	DFB	Diff. value	−0.147	−0.164	0.035	0.055	0.187	0.288
		%	−13.89%	−12.42%	2.30%	3.13%	10.72%	12.88%
	AUC	Diff. value	−0.086	−0.097	0.108	0.133	0.029	0.036
		%	−7.67%	−7.00%	6.87%	7.17%	1.82%	1.80%
DFB Compared to	AUC	Diff. value	0.061	0.067	0.074	0.077	−0.158	−0.252
		%	5.46%	4.82%	4.67%	4.17%	−9.96%	−12.73%

**Table 10:** Cross-validation results for the AUC-DFB version, all values in mmol/L. The V1 version is the model trained with the training/validation data division as described above, while the V2 and V3 versions use another third of the samples for validation.

	Prediction Horizon			
	120 min		180 min	
	MAE	RMSE	MAE	RMSE
AUC-DFB (V1)	1.47	1.718	1.562	1.946
V2	1.519	1.731	1.506	2.092
V3	1.408	1.702	1.603	2.014

## Data Availability

The detailed, anonymized data sets used to train the neural network are available as Supplementary Materials Table S1 to this article and as a public Mendeley Data dataset. Each meal record contains the meal’s dietary content, the computed absorption parameters, and the CGM data used for training or validation of the NN, such that the results reported in this paper can be fully reproduced.

## Supplementary Materials

The following are available online at https://www.mdpi.com/article/10.3390/medicina57070676/s1, Table S1: Train_valid_data_AUC_DFB.
